# Wellbeing, alcohol use and sexual activity in young teenagers: findings from a cross-sectional survey in school children in North West England

**DOI:** 10.1186/1747-597X-5-27

**Published:** 2010-11-10

**Authors:** Penelope A Phillips-Howard, Mark A Bellis, Linford B Briant, Hayley Jones, Jennifer Downing, Imogen E Kelly, Timothy Bird, Penny A Cook

**Affiliations:** 1 Centre for Public Health, Research Directorate, Liverpool John Moores University, Henry Cotton Campus, 15-21 Webster Street, Liverpool L3 2ET, UK; 2 Department of Engineering Mathematics, University Of Bristol, Queen's Building, University Walk, Bristol, BS8 1TR, UK; 3 Royal Manchester Children's Hospital, Oxford Road, Manchester M13 9WL, UK; 4 School of Psychological Sciences, Coupland 1, University of Manchester, Oxford Road, Manchester, M13 9PL, UK

## Abstract

**Background:**

Adolescent health is a growing concern. High rates of binge drinking and teenage pregnancies, documented in the UK, are two measures defining poor wellbeing. Improving wellbeing through schools is a priority but information on the impact of wellbeing on alcohol use, and on sexual activity among schoolchildren is limited.

**Methods:**

A cross-sectional survey using self-completed questionnaires was conducted among 3,641 schoolchildren aged 11-14 years due to participate in a sex and relationships education pilot programme in 15 high schools in North West England. Bivariate and multivariate analyses were conducted to examine the relationship between wellbeing and alcohol use, and wellbeing and sexual activity.

**Results:**

A third of 11 year olds, rising to two-thirds of 14 year olds, had drunk alcohol. Children with positive school wellbeing had lower odds of ever drinking alcohol, drinking often, engaging in any sexual activity, and of having sex. General wellbeing had a smaller effect. The strength of the association between alcohol use and the prevalence of sexual activity in 13-14 year olds, increased incrementally with the higher frequency of alcohol use. Children drinking once a week or more had 12-fold higher odds of any sexual activity, and 10-fold higher odds of having sex. Rare and occasional drinkers had a significantly higher odds compared with non-drinkers.

**Conclusions:**

The relationship between wellbeing and alcohol use, and wellbeing and sexual activity reinforces the importance of initiatives that enhance positive wellbeing in schoolchildren. The association between alcohol use and sexual activity highlights the need for integrated public health programmes. Policies restricting alcohol use may help reduce sexual exposure among young teenagers.

## Background

The health of adolescents is a growing concern in many countries, particularly in the UK,[1] where children have been identified as having one of the lowest wellbeing scores amongst wealthy nations[2,3]. Comparison between countries of the Organisation for Economic Co-operation and Development revealed the lower than average overall child wellbeing score reflected measures on risky behaviours (binge drinking and teen pregnancies), and higher rates of 15-19 year olds not in education, training or employment[4]. As such, the rising prevalence of adolescent alcohol misuse and stubbornly high teenage pregnancy rates are recognised to be national public health priorities[5-8]. Early alcohol use increases the risk of dependency and fuels the growing burden of alcohol-related disease[5]. The teenage birth rate has been found to most closely relate to the overall index of wellbeing[3].

An array of government programmes have been developed to enhance the health and wellbeing of children and young people in the UK, culminating with the Healthy Child Programme (HCP) launched by the Department of Health and Department for Children, Schools and Families[8]. Through the HCP, schools are charged with the duty to promote the wellbeing of their children, provide healthy food, and deliver comprehensive personal, social and health (PSHE) education, including sex and relationships education (SRE). Monitoring of success, through Ofsted,[9] will focus on the five 'Every Child Matters' outcomes: be healthy, stay safe, enjoy and achieve, make a positive contribution, and achieve economic wellbeing[10]. Schools are encouraged to develop their monitoring criteria based on local 'vital signs'; while a number of indicators are recommended for alcohol, no information is available on young people's sexual activities. Evidence demonstrating the relationship between wellbeing indicators and risky health behaviour is limited, particularly among young teenagers, who seldom participate in sexual health research. Research suggests dislike of school is an important determinant of teen pregnancy,[11,12] and poor school engagement is associated with binge drinking[13]. An association between wellbeing and alcohol use, and wellbeing and sexual activity is therefore likely with important connotations for health policy.

We present findings from a baseline study piloting a sex and relationships (SRE) package for Year 7 to 9 (11-14 year old) children in high schools in North West England, a region documented to have high rates of teenage binge drinking,[14] and above average teenage pregnancy rates[15]. The questioning of children pre-intervention provided a unique opportunity to generate measures on their general and school wellbeing, in association with their reported use of alcohol and their sexual activities.

## Methods

Of 17 schools recruited by Government Office North West (GONW) to participate in the teacher training and dissemination of the pilot SRE package, 15 contributed to the pre-intervention baseline cross-sectional survey in the target population. All children in Years 7 to 9 (aged 11 to 14 years), in 66 classes selected by the school leads to participate in the pilot SRE programme, were eligible in the sampling frame. Children outside the year group, severely incapacitated, did not speak English, had a signed withdrawal from consent by a parent or carer, or who personally did not assent, were excluded from the survey.

A baseline cross-sectional survey was organized by staff given lead responsibility for the pilot SRE programme at each local authority in the autumn term of 2008. Letters of invitation were sent to children's homes, to inform parents and enable them to opt-out prior to the survey. Self-completion of the closed questionnaires took place during a PSHE lesson after teachers discussed the project with children and witnessed written assent. The questionnaire asked children about their attitudes towards school life, general wellbeing, and activities outside school including their use of alcohol. Year 9 were also asked about their sexual behaviour. Children were advised they did not have to answer questions and they could stop answering at any time. Teachers or school nurses were available to talk or signpost children if needed.

The study and tools were submitted to and approved by the Ethics Committee of Liverpool John Moores University and the study was endorsed by the Regional Safeguarding Office. Data were stored in an anonymised form.

Data analyses were conducted using SPSS for Windows (Release v17.0). Wellbeing questions were abstracted from the national Canadian Youth, Sexual Health, and HIV/AIDS Study [16]. Five items were incorporated on school wellbeing and eight items were incorporated on general wellbeing, three of which were ambiguous and excluded from analyses (see Table 1). Wellbeing items were collapsed from the original 5-point Likert scale into 3-points (agree/don't know/disagree). Other characteristics were binary (yes/no). Alcohol endpoints comprised 'ever drunk' (a full drink not just a sip) and whether those who had ever consumed alcohol were drinking seldom (<once/week) or often (≥once a week). Frequency of alcohol consumption was also included as an independent variable in multivariate analyses of sexual activity and sex, grouped into never, rare (<once/month), occasional (≥once month or more), and frequent (≥once a week). Sexual activity comprised kissing, deep kissing, sexual petting, oral sex, or sexual intercourse. Definitions for each were incorporated into the question. Sex represented a 'yes' response to having had oral sex or intercourse. Bi- and multivariate associations between wellbeing exposure indicators and endpoints (alcohol use, drink often, sexual activity and sex) are reported with unadjusted and adjusted odds ratios, 95% confidence intervals. We included one set of wellbeing exposure indicators in the multivariate regression model. The school wellbeing indicator (school is a nice place to be) was chosen because of prior reference to its potential association with teenage pregnancy[11]. The general wellbeing indicator (you have a happy home life) was selected because of its use in school monitoring[9]. Clustering of responses by school was accounted for using the Complex Samples module in SPSS (Release v17.0). This uses Taylor series linearization methods to estimate variances that are in turn used to develop correct standard errors and confidence intervals for statistics of interest. Age and gender were controlled for in the regression models. Differences between groups were determined using Pearson's χ^2 ^test, and significance assigned at the 5% level.

**Table 1:** Bivariate associations between wellbeing and alcohol use in schoolchildren aged 11 to 14 years

Wellbeing indicators^		Ever Alcohol #			Drink alcohol often $		
**(a) General Wellbeing**		Wald F*	**N (%yes)**	**OR (95% CI)**	**Wald F**	**N(%yes)**	**OR (95% CI)**

You would like to change the way you look	Disagree	7.028	1441 (39)	1	2.338	409 (55)	1
	Don't know		844 (46)	1.36 (1.09-1.68)		283 (52)	0.91 (0.68-1.20)
	Agree		1171 (53)	1.77 (1.27-2.48)		472 (61)	1.27 (0.88-1.83)
You can talk openly with your parents about your problems	Agree	9.270	2550 (42)	1	5.268	804 (54)	1
	Don't know		483 (53)	1.52 (1.17-1.98)		174 (64)	1.54 (1.10-2.15)
	Disagree		415 (59)	1.94 (1.33-2.83)		177 (63)	1.43 (1.03-2.00)
You are able to assert your views among your friends	Agree	6.181	2420 (48)	1	0.853	854 (55)	1
	Don't know		737 (41)	0.77 (0.65-0.90)		214 (62)	1.30 (0.83-2.04)
	Disagree		250 (46)	0.95 (0.72-1.26)		79 (56)	1.02 (0.76-1.37)
You are often sorry for things that you do ('remorse')	Disagree	1.963	553 (45)	1	2.075	189 (65)	1
	Don't know		828 (48)	1.16 (0.90-0.50)		272 (54)	0.62 (0.37-1.03)
	Agree		2055 (45)	1.03 (0.75-1.41)		696 (56)	0.67 (0.44-1.01)
You have a happy home life	Agree	32.899	2953 (44)	1	4.696	955 (55)	1
	Don't know		273 (55)	1.52 (1.35-1.72)		115 (63)	1.39 (1.03-1.87)
	Disagree		216 (57)	1.64 (1.23-2.19)		84 (69)	1.85 (1.17-2.92)

**(b) School Wellbeing**							

Your teachers treat you fairly	Agree	13.041	2757 (42)	1	11.091	872 (53)	1
	Don't know		477 (55)	1.68 (1.11-2.56)		177 (63)	1.52 (1.04-2.21)
	Disagree		247 (67)	2.81 (1.85-4.27)		120 (72)	2.22 (1.37-3.62)
Your school is a nice place to be	Agree	20.690	2443 (43)	1	2.263	768 (54)	1
	Don't know		663 (51)	1.39 (1.18-1.65)		251 (59)	1.22 (0.87-1.71)
	Disagree		369 (58)	1.88 (1.52-2.33)		146 (64)	1.47 (0.94-2.29)
In your school, students take part in making the rules	Agree	9.174	1313 (43)	1	6.677	415 (55)	1
	Don't know		1249 (44)	1.02 (0.78-1.33)		386 (52)	0.90 (0.61-1.33)
	Disagree		873 (53)	1.51 (1.17-1.95)		355 (63)	1.37 (0.93-2.01)
Students are treated too strictly/severely in your school	Disagree	27.114	1120 (38)	1	2.443	336 (57)	1
	Don't know		1349 (45)	1.37 (1.15-1.62)		433 (53)	0.85 (0.61-1.20)
	Agree		978 (56)	2.12 (1.72-2.61)		390 (60)	1.14 (0.82-1.58)
Teachers expect too much of you	Disagree	15.394	1021 (37)	1	2.492	310 (53)	1
	Don't know		1076 (42)	1.23 (0.98-1.55)		340 (55)	1.08 (0.72-1.61)
	Agree		1337 (55)	2.05 (1.52-2.77)		503 (60)	1.35 (0.98-1.85)

^general and school wellbeing indicators derived from 5-point Likert Scale collapsed into agree/disagree/don't know;

# 'have you ever drunk alcohol; a full drink, not just a sip';

$ 'drink alcohol often' collapsed into seldom (<once a week); and often (≥once a week); students reporting 'never' or don't know how often they drink are excluded;

OR: Odds Ratio (95% confidence intervals; CI); 1 = referent.

*The complex samples procedure produced Wald F-values with a pair of degrees of freedom (df) values (df2, df13).

## Results

The 15 schools successfully enrolled 3,641 children from 66 classes into the cross-sectional survey at baseline. Parental withdrawal forms were received for 30 children, a further 65 children refused assent, and 255 questionnaires returned were unused/spoilt, generating a non-response rate of 10%. Respondents were equally split by gender (51.6% female). More Year 7 classes were enrolled into the SRE pilot programme, generating a higher proportion of responses (1430, 39.3%) compared with Year 8 (1204; 33.1%) and Year 9 (1007; 27.7%).

### Associations between wellbeing and reported alcohol use

Overall, 45.5% of children reported ever drinking alcohol. Prevalence increased from 32.0% in 11 year olds to 65.8% in 14 year olds (Pearsons χ^2 ^= 190.32, df3, p < 0.001). Significantly more 11 year old boys than girls had drunk alcohol (39% versus 25% (Pearsons χ^2 ^= 9.58, df1, p < 0.001), leveling out by age 14 (66.9% in girls versus 63.5% in boys). The mean age of reported first drink for 11 year old boys and girls was 9.4 (standard deviation SD 2.3) and 10.0 (SD 1.9), respectively (p < 0.001). Among drinkers, there was a significant trend for increased frequency of drinking by age (Pearsons χ^2 ^= 11.711, df3, p = 0.008; Linear by linear association χ^2 ^= 6.411, df1, p = 0.011).

In bivariate analyses, the odds of ever drinking alcohol were two-fold higher in children responding negatively to school wellbeing indicators (Table 1). Indicators depicting children's relationship with teachers were more strongly associated. Among general wellbeing indicators, children disagreeing they had a happy home life, or disagreed they were able to talk to parents about problems had the highest odds of ever drinking. Similar associations were recorded between drinking often (versus seldom, among drinkers) and wellbeing (Table 1). In multivariate analysis age, but not gender, was strongly significant for ever drinking, and weakly associated with drinking often (Table 2). A negative response to the selected school wellbeing statements (school is a nice place to be) was associated with a 53% higher odds for ever drinking (Table 2). A negative response to general wellbeing (have a happy home life) was associated with 78% higher odds of drinking often (Table 2).

**Table 2:** Multivariate associations between wellbeing, alcohol use and sexual activity in school-children+

		Alcohol use endpoints						Sexual activity endpoints					
		**Ever drunk alcohol#** **N = 3286**			**Drink often** **N = 1096$**			**Any sexual activity~** **N = 679**			**Sex~~** **N = 666**		

		**Wald** **[df]**	**%**	**AOR (95% CI)**	**Wald** **[df]**	**%**	**AOR (95% CI)**	**Wald** **[df]**	**%**	**AOR (95% CI)**	**Wald** **[df]**	**%**	**AOR (95% CI)**

Gender	Male	1.665 [1]	47	1	0.066 [1]	57	1	3.500 [1]	64	1	4.829 [1]	18	1
	Female		44	0.88 (0.70-1.09)		56	0.98 (0.79-1.20)		56	0.71 (0.47-1.07)		11	0.52 (0.27-1.01)

Age	11	21.732 [3]	33	1	10.618 [3]	56	1	-	-	-	-	-	-
	12		42	1.44 (1.12-1.85)		51	0.80 (0.59-1.08)	-	-	-	-	-	-
	13		59	2.91 (1.75-4.82)		59	1.07 (0.85-1.34)	0.175 [1]	59	1	0.294 [1]	13	1
	14		66	3.86 (1.66-8.93)		68	1.65 (0.98-2.77)		61	1.07 (0.74-1.56)		16	1.22 (0.54-2.72)

Alcohol frequency β	Never	-	-	-	-	-	-	51.288 [3]	28	1	60.447 [3]	4	1
	Rare	-	-	-	-	-	-		63	4.50 (2.18-9.29)		7	1.82 (0.64-5.14)
	Occasional	-	-	-	-	-	-		75	6.77 (3.13-14.63)		16	4.37 (1.41-13.55)
	Frequent	-	-	-	-	-	-		83	12.68 (5.52-29.12)		30	10.84 (4.85-30.53)

WB^ School is a nice place to be	Agree	21.363 [2]	42	1	3.766 [2]	54	1	15.823 [2]	54	1	6.753 [2]	11	1
	Disagree		58	1.53 (1.20-1.95)		65	1.40 (0.95-2.07)		75	2.56 (1.51-4.33)		20	1.86 (0.82-4.23)
	Don't know		51	1.32 (1.11-1.57)		59	1.18 (0.86-1.61)		64	1.55 (0.86-2.81)		18	1.93 (1.09-3.41)
WB^ You have a happy home life	Agree	29.521 [2]	44	1	7.219 [2]	55	1	5.171 [2]	58	1	2.133 [2]	14	1
	Disagree		56	1.40 (1.03-1.89)		70	1.78 (1.10-2.90)		68	1.31 (0.53-3.20)		21	1.36 (0.81-2.27)
	Don't know		55	1.39 (1.21-1.59)		63	1.37 (0.92-2.05)		69	1.81 (1.01-3.27)		11	0.83 (0.23-2.98)

+ sample for alcohol include school-children in Years 7 to 9 (11 to 14 years); sample for sexual endpoints restricted to Year 9 (13-14 years);

# ever drunk alcohol (a full drink, not just a sip); % = yes;

$ 'drink alcohol often' collapsed into seldom (<once/week); and often (≥once/week). Students reporting 'never' or don't know how often they drink are excluded;

~ any sexual activity includes kissing, deep kissing, petting, oral sex, sexual intercourse; ~~Sex is limited to oral sex and/or intercourse;

β Alcohol frequency: never, rare (<once/month), occasional (≥once/month or more), frequent (≥once/week);

WB^ selected example wellbeing indicators for school and general wellbeing (see methods);

AOR: Adjusted Odds Ratio (95% confidence intervals; CI); 1 = referent.

*The complex sample procedure produced Wald chi-square values with a single degree of freedom [df] value

### Associations between wellbeing and sexual activity (Year 9)

Of 768 children in Year 9 (mean age 13.25 years), an equal proportion of boys (49.4%) and girls (55.2%) reported having had some type of sexual activity (Pearsons χ^2 ^= 2.521, df1, p = 0.112). Over half reported kissing, with slightly fewer reporting deep kissing, and a quarter had petted. Oral sex was reported by 11% (boys 13.9%, girls 8.7%; Pearsons χ^2 ^= 5.148, df1, p = 0.023) and intercourse by 10.2% (boys 13.0%, girls 8.0%; Pearsons χ^2 ^= 5.083, df1, p = 0.024).

In bivariate analyses, having any sexual activity, and having sex, was significantly associated with negative responses to school wellbeing indicators (Additional file 1, Table S1). However, negative responses to general wellbeing indicators were mostly not statistically associated. In multivariate analyses, a negative response to school wellbeing (school is a nice place to be) was associated with a two and a half-fold higher odds of having any sexual activity (Table 2). An increased odds of having sex if male was non-significant. The odds of having sex was higher among children responding negatively to school and general wellbeing indicators, however, these did not reach statistical significance.

### Associations between alcohol and sexual activity (Year 9)

**Figure 1:**
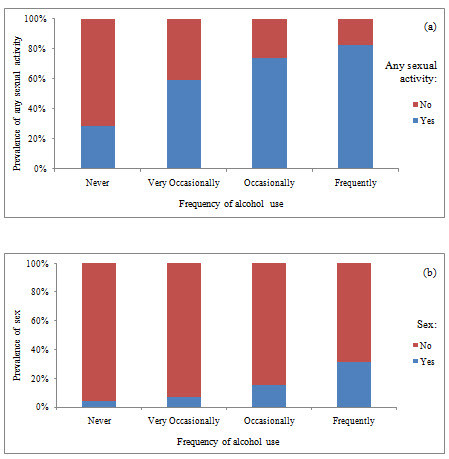
**The prevalence of a) any sexual activity and b) sexual intercourse by reported frequency of alcohol use (n = 708)**. Alcohol frequency: never, very occasionally (rare) (<once/month), occasionally (≥once/month or more), frequently (≥once/week); (a) Any sexual activity includes kissing, deep kissing, petting, oral sex, sexual intercourse; Statistics for any sexual activity: Pearsons χ^2 ^= 142.31, df3, p < 0.001; Linear-by-Linear Association χ^2 ^= 133.41, df1, p < 0.001 (b) Sexual intercourse is limited to those reporting having oral sex and/or intercourse; Statistics for sexual intercourse: Pearsons χ^2 ^= 69.48, df3, p < 0.001; Linear-by-Linear Association χ^2 ^= 63.68, df1, p < 0.001.

The prevalence of reported sexual activity was associated with their reported frequency of drinking alcohol (Figure 1). The prevalence of sexual activity rose significantly in students who drank more frequently (Pearsons χ^2 ^= 142.31, df3, p < 0.001; Linear-by-Linear Association χ^2 ^= 133.41, df1, p < 0.001). A similar highly significant trend was found for the prevalence of sex in students who drank more frequently (Pearsons χ^2 ^= 69.48, df3, p < 0.001; Linear-by-Linear Association χ^2 ^= 63.68, df1, p < 0.001).

In multivariate analysis, the odds of having any sexual activity, and of having sex was incrementally associated with alcohol use, with the odds rising as the frequency of drinking increased (Table 2). This increased from a four-fold higher odds of having any sexual activity in rare compared with non-drinkers, to six-fold higher odds in occasional drinkers, while those drinking weekly or more had a twelve-fold higher odds of any sexual activity compared with non-drinkers (Table 2). Similar rising odds were evident between drinking frequency and having sex. Rare drinkers had two-fold higher odds, occasional drinkers had four-fold higher odds, and frequent drinkers had ten-fold higher odds of having sex (Table 2).

## Discussion

Our study provides evidence that negative subjective wellbeing is associated with increased odds of drinking alcohol and engagement with sexual activity. While cause and effect cannot be extrapolated from cross-sectional surveys, the association between wellbeing and risk behaviours was incremental suggesting--as a minimum--that children with poor wellbeing are more likely to also encounter behavioural health risks. Generation of internationally recognised key indicators of poor child wellbeing, such as income and poverty,[3] was precluded from this school-based study since children could not accurately record these data. Nevertheless, youth resilience, self-esteem, social- and school-connectedness are critical determinants of successful transition from childhood into adulthood,[17-19] and the general and school wellbeing indicators we chose represent these themes[16]. Data were generated from the baseline of a sex and relationship education project. Schools were chosen by the Government Office North West, rather than random selection from a broader schools list. The sample was not intended to be representative but opportunistic for both students and classroom participation. Survey participants may thus not be fully representative of all children in North West England. However, schools would have included those agreeing to participate in the SRE pilot, characterizing 'progressive' teaching attitudes, as well as others representing schools in need of educational change. While children across the wellbeing spectrum were represented in our study, and analyses achieved statistical significance, generalising results directly to wider populations should be undertaken with caution. Our main analyses thus focuses on relationships between variables recorded by individual participants and do not seek to establish population prevalence. Despite these caveats we believe this study contributes further evidence to the hypothesis that school wellbeing influences children's risk behaviours,[11,12,20] and supports current efforts to enhance wellbeing at school[8].

Dislike of school has been identified as a potential contributor to teenage pregnancy risk,[11,12] and to substance abuse, poor mental health, and low academic achievements[13,17,19]. Strengthening school-connectedness and development of resiliency programmes has thus been recommended to reduce teen pregnancies and substance misuse in the UK[13,20]. However, a UK-based multi-component youth development programme did not secure a reduction in teen pregnancy, substance abuse and other harms, suggesting further understanding is needed to clarify factors that are associated with risky behaviours in teenagers[21]. Our study found children stating a dislike of school had 2.5-fold higher odds of having any sexual relationship and 86% higher odds of having sex, although the latter was not statistically significant. Dislike of school also strongly predicted alcohol use. We also explored other school wellbeing indicators, noting teacher-related markers were more strongly associated with alcohol use and frequency of drinking. Clearly, children involved with risky health behaviours are most in need of guidance and support through school programmes, but they appear to be the very children who poorly engage and are thus less receptive to learning new skills. Programmes supporting targeted activities to bring about healthier behaviours, such as the Healthy Schools enhancement model,[8] thus need to pay particular attention to fostering the engagement of disenfranchised children.

We report evidence of an incremental association between the frequency of alcohol use and the prevalence of sexual activity, including having sex, among young teenagers. To our knowledge, this is the first study to show such a strong and consistent association between alcohol use and sexual activity in children the UK, although the contributions of alcohol to pregnancy has been explored[22]. In the largest UK-based study of sexual activity in teenagers, being drunk or stoned elevated the risk of regretted sex, but student self-recording of being drunk was not found to be a strong predictor of sexual activity[23]. However, a survey among 15-16 year olds in north west England in 2007 found an association between binge drinking and sexual regret, but data on children's sexual activity were not available to explore this association further[14]. International studies have noted associations between alcohol and early onset of sexual activity,[24,25] having multiple sexual partners,[26,27] and becoming pregnant[28]. An American study of young people aged 12 to 20 years, showed a correlation between pregnancy and rates of binge drinking[29]. They documented a 30-fold higher odds of making/becoming pregnant in persons bingeing up to 10 times in the past 30 days, 10-fold in those bingeing twice, and 4-fold in non-bingers, compared with non-drinkers. In our study we also noted that the odds of having sexual activity (and sex) were higher among children who reported drinking rarely, compared with non-drinkers. This corroborates studies showing that even low level drinking increases the potential risk of harm[14,29].

## Conclusions

This study adds UK data to an international body of knowledge showing an association between alcohol and sexual activity, and confirms this occurrence in children, including young teenagers exploring early sexual activity prior to sexual debut. Such findings, along with evidence showing early onset of alcohol use, highlight the need to start educational activities early in schoolchildren's lives and calls into question the wisdom of abandoning statutory sex and relationships education. The relationship between risk behaviours underscores the need to integrate public health strategies and policies, to maximise opportunities to combat harms associated with alcohol abuse and poor sexual health. It also supports calls to strengthen policies to reduce the access and availability of alcohol to young teenagers[5,6,14]. The association between risk behaviours and wellbeing reinforces the importance of wellbeing and life skills programmes developed for school-aged children. Information generated from our survey provides early insights on the potential role of wellbeing in modulating risk behaviours that need to be tested more widely if we are to understand and develop meaningful strategies to protect children and young people from harm.

## Competing interests

The authors declare that they have no competing interests.

## Authors' contributions

PAPH conceptualised, oversaw and wrote the study for this manuscript. PAPH, MAB and LBB analysed the data. HJ, IEK, TB and PAC contributed to data preparation, analysis and manuscript production. PAC, MAB, and JD designed the SRE evaluation project and edited the manuscript. HJ and JD managed the SRE evaluation project. MAB appraised the study for intellectual content. All authors read and approved the final manuscript.

## Supplementary Material

Additional file 1 **Table S1: Bivariate associations between wellbeing and sexual activity among schoolchildren aged 13 to 14 years**. Click here for file
