# Supplementary material for: Wellbeing, alcohol use and sexual activity in young teenagers: findings from a cross-sectional survey in school children in North West England
Source: Subst Abuse Treat Prev Policy. 2010 Nov 10;5:27. doi: 10.1186/1747-597X-5-27 (PMC2993686; doi:10.1186/1747-597X-5-27)
Supplement: Additional file 1 — Table S1: Bivariate associations between wellbeing and sexual activity among schoolchildren aged 13 to 14 years. [file 1747-597X-5-27-S1.DOC]

Additional file Table S1: Bivariate associations between wellbeing and sexual activity among schoolchildren aged 13 to 14 years

| Wellbeing indicators^ |  | Any sexual activity~ | | | Sex~~ | | |
| --- | --- | --- | --- | --- | --- | --- | --- |
| (a) General Wellbeing |  | Wald F* | N (%yes) | OR (95% CI) | Wald F | N (%yes) | OR (95% CI) |
| You would like to change the way you look | Disagree | 0.440 | 286 (57) | 1 | 4.594 | 279 (15) | 1 |
|  | Don’t know |  | 178 (56) | 0.95 (0.57-1.60) |  | 175 (10) | 0.59 (0.40-0.88) |
|  | Agree |  | 301 (62) | 1.19 (0.74-1.90) |  | 293 (15) | 0.94 (0.56-1.60) |
| You can talk openly with your parents about your problems | Agree | 1.507 | 533 (56) | 1 | 2.280 | 524(11) | 1 |
|  | Don’t know |  | 99(63) | 1.31 (0.81-2.13) |  | 94 (19) | 1.83 (0.99-3.41) |
|  | Disagree |  | 129 (65) | 1.46 (0.91-2.36) |  | 125 (18) | 1.74 (0.80-3.80) |
| You are able to assert your views among your friends | Agree | 2.265 | 575 (61) | 1 | 0.985 | 563 (14) | 1 |
|  | Don’t know |  | 137(50) | 0.63 (0.40-1.01) |  | 134 (10) | 0.65 (0.28-1.52) |
|  | Disagree |  | 41 (59) | 0.91 (0.51-1.63) |  | 39 (18) | 1.32 (0.75-2.34) |
| You are often sorry for things that you do (‘remorse’) | Disagree | 0.037 | 147 (58) | 1 | 4.065 | 142 (20) | 1 |
|  | Don’t know |  | 180(58) | 1.00 (0.76-1.32) |  | 177 (10) | 0.49 (0.27-0.88) |
|  | Agree |  | 431 (59) | 1.04 (0.78-1.37) |  | 421 (12) | 0.55 (0.29-1.05) |
| You have a happy home life | Agree | 3.057 | 621 (57) | 1 | 3.455 | 606 (13) | 1 |
|  | Don’t know |  | 64(69) | 1.68 (1.00-2.84) |  | 63 (11) | 0.83 (0.29-2.39) |
|  | Disagree |  | 62 (69) | 1.73 (0.99-3.04) |  | 61 (21) | 1.81 (1.09-3.01) |
| (b) School Wellbeing | | | | | | | |
| Your teachers treat you fairly | Agree | 7.167 | 569 (55) | 1 | 7.938 | 558 (12) | 1 |
|  | Don’t know |  | 108 (69) | 1.81 (0.87-3.73) |  | 102 (15) | 1.31 (0.66-2.60) |
|  | Disagree |  | 86 (72) | 2.14 (1.39-3.30) |  | 85 (26) | 2.65 (1.55-4.52) |
| Your school is a nice place to be | Agree | 16.925 | 499 (54) | 1 | 4.406 | 490 (11) | 1 |
|  | Don’t know |  | 145 (64) | 1.52 (0.97-2.38) |  | 139 (18) | 1.73 (1.05-2.87) |
|  | Disagree |  | 120 (71) | 2.06 (1.49-2.85) |  | 118 (19) | 1.81 (1.16-2.84) |
| In your school, students take part in making the rules | Agree | 1.913 | 265 (51) | 1 | 5.084 | 260 (9) | 1 |
|  | Don’t know |  | 249 (62) | 1.59 (0.94-2.68) |  | 244 (12) | 1.33 (0.83-2.12) |
|  | Disagree |  | 246 (65) | 1.79 (0.92-3.46) |  | 238 (21) | 2.55 (1.32-4.94) |
| Students are treated too strictly/severely in your school | Disagree | 4.936 | 259 (51) | 1 | 2.415 | 254 (10) | 1 |
|  | Don’t know |  | 288 (59) | 1.39 (0.98-1.96) |  | 281 (14) | 1.46 (0.86-2.45) |
|  | Agree |  | 215 (68) | 2.11 (1.24-3.61) |  | 209 (17) | 1.83 (1.00-3.34) |
| Teachers expect too much of you | Disagree | 3.695 | 226 (51) | 1 | 4.605 | 220 (7) | 1 |
|  | Don’t know |  | 226 (60) | 1.46 (0.86-2.47) |  | 222 (14) | 2.14 (1.00-4.55) |
|  | Agree |  | 308 (64) | 1.74 (1.13-2.67) |  | 300 (19) | 3.28 (1.40-7.65) |

**^** wellbeing indicators derived from 5-point Likert Scale collapsed into agree/disagree/don’t know;

~ any sexual activity includes kissing, deep kissing, petting, oral sex, sexual intercourse;

~~Sex is limited to only those reporting oral sex and intercourse

OR: Odds Ratio (95% confidence intervals; CI); 1= referent.

* The complex samples procedure produced Wald F-values with a pair of degrees of freedom (df) values (df2, df9).
